# Sociodemographic, Behavioral and Oral Health Factors in Maternal and Child Health: An Interventional and Associative Study from the Network Perspective

**DOI:** 10.3390/ijerph18083895

**Published:** 2021-04-08

**Authors:** Juliana Ribeiro Francelino Sampaio, Suely Arruda Vidal, Paulo Savio Angeiras de Goes, Paulo Felipe R. Bandeira, José Eulálio Cabral Filho

**Affiliations:** 1 Faculty of CECAPE, Medicine College Estácio of Juazeiro do Norte, Juazeiro do Norte, CE 63040-360, Brazil; 2 Study Group on Health Management and Evaluation at IMIP/Pernambuco, Institute of Integral Medicine Fernando Figueira—IMIP, Recife, PE 50070-550, Brazil; suely@imip.org.br; 3 Post-Graduation Stricto Sensu, Institute of Integral Medicine Professor Fernan-do Figueira—IMIP, Recife, PE 50070-550, Brazil; eulalio@imip.org.br; 4 Child and Adolescent Health Program, Department of Clinic and Preventive Dentistry, Federal University of Pernambuco—UFPE, Recife, PE 50670-901, Brazil; paulosaviogoes@gmail.com; 5 Center for Biological and Health Sciences, Regional University of Cariri-URCA/Ceará, Northeastern Family Health Training Network—RENASF, Crato, CE 63105-000, Brazil; paulo.bandeira@urca.br; 6 Brazilian Journal of Maternal and Child Health of IMIP, Recife, PE 50070-550, Brazil

**Keywords:** integrality in health, oral health, prenatal care, clinical trial, network perspective

## Abstract

Oral healthcare during pregnancy needs to be part of the assistance routine given to pregnant women by health professionals as a way to encourage self-care and strengthen the general health of the mother and the baby. The aim of this study was to evaluate the effectiveness of an integrated oral healthcare intervention for pregnant women and to analyze the association of sociodemographic, behavioral, oral health and general maternal and child health factors in prenatal care at usual risk in primary care in a city in the northeast of Brazil, in 2018–2019. A controlled, randomized, single-blinded community trial was conducted. The intervention group (IG) received dental assistance and collective health education actions in conversation circles, while the control group (CG) received the usual assistance. All pregnant women (146 in total, 58 from IG and 88 from CG) that took part in the trial answered a questionnaire and underwent a dental examination at the beginning of prenatal care and at the puerperal visit. To assess the effect of the intervention, a network analysis was used. The results have shown that being in the control group was associated with neonatal complications (0.89) and prematurity (0.54); the use of tobacco and alcohol are associated with high risk in initial and final oral health; lower educational level of the pregnant women implicates high risk for initial oral health (−0.19), final oral health (−0.26), pregnancy complications (−0.13), low birth weight (−0.23), prematurity (−0.19) and complications in the newborn (−0.14). Having a low family income (≤261.36 USD) has shown a high risk for initial oral health (−0.14), final oral health (−0.20) and prematurity (−0.15). The intervention based on integrated oral healthcare for pregnant women indicated that socioeconomic and behavioral factors must be considered as determinants for the quality of women and children’s health and that multi-professional performance during prenatal care contributes to the positive outcomes of pregnancy.

## 1. Introduction

Pregnancy promotes natural organic alterations and changes in the oral cavity, imposing on health professionals the need for a differentiated approach in prenatal care [1].

Female oral health is often disregarded during pregnancy by professionals who perform prenatal care and even by dentists, because they believe that dental treatment can harm the fetus [2]. Pregnant women, because of unawareness or uncertainty, difficulty in access, beliefs and fear, do not seek the necessary treatment, thus contributing to oral health problems [3,4].

A study carried out in Italy on oral hygiene practices and postpartum oral health conditions showed that although 99.1% of pregnant women brushed their teeth every day, 59.9% visited the dentist annually, with an average score of caries experience, decayed, lost and filling permanent teeth (DLFT), equal to eight and severe periodontal disease in 21.9% of them [5]. These results highlight the importance of professionals who work directly in prenatal care, obstetricians and nurses, collaborating with dentists to encourage pregnant women to adhere to oral hygiene recommendations, appropriate brushing techniques and visits to the dentist [1,6].

Prospective studies [7,8] and a clinical trial [9] analyzing the relationship between periodontal disease in pregnant women and prematurity and low birth weight have pointed to a possible risk factor. However, there is a need for further studies, including multicenter studies, to reinforce this statement.

Integrated care for patients, centered on the person and considering individual needs, should guide a new model of primary care practice in oral health. It requires the adoption of a work process that incorporates the health situation of the population belonging to the geographic territory under the responsibility of the health team into planning and practices, thus strengthening the community [10,11].

Besides good oral hygiene habits, gestational outcomes may be influenced by behavioral factors such as smoking habits and social determinants (educational level and income); the interaction between these factors characterizes a complex system, since these variables have different natures with different units of measurement and come from different intervention areas [12,13]. In this sense, complex systems can be better understood based on network analysis, which allows the evaluation of multiple interactions, most often nonlinear, between variables [14]. Network analysis also allows us to understand the role of each variable in a system, indicating those that are most sensitive to interventions and those that are most rapidly affected by these interventions [15,16]. This is particularly important to optimize both care and intervention in health research.

The present study aimed to assess the effectiveness of an integrated oral healthcare intervention for pregnant women and to verify the association of sociodemographic, behavioral and oral health factors in maternal and child health in the groups (intervention and control).

## 2. Materials and Methods

A controlled, randomized, single-blinded trial was carried out with usual-risk pregnant women. The study was conducted in a municipality in northeastern Brazil with a population of 271,926 inhabitants, a Municipal Human Development Index (MHDI) score of 0.694 and divided administratively into seven health districts. Registro Brasileiro de Ensaios Clínicos (Brazilian Registry of Clinical Trials) (ReBEC): RBR-649bhb.

### 2.1. Sample Selection and Group Allocation

**Figure 1:**
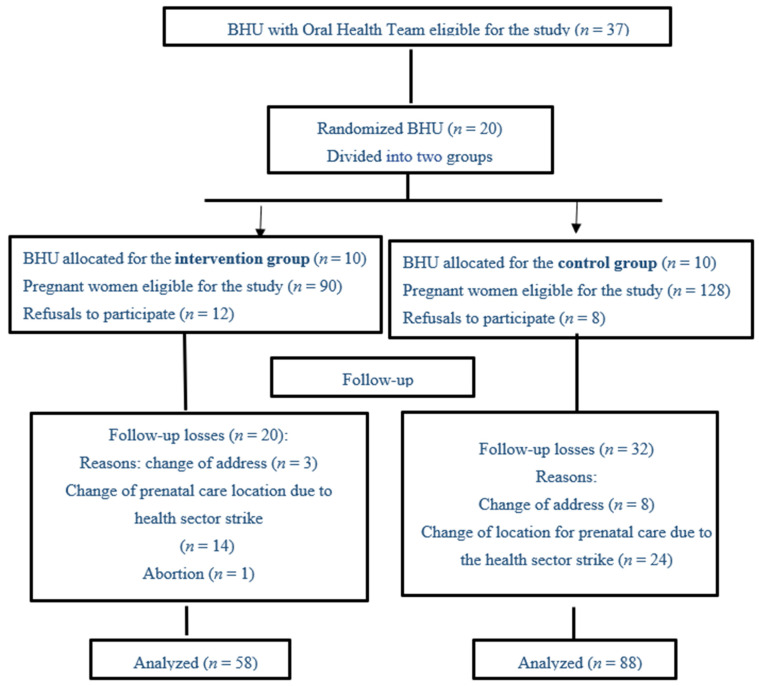
CONSORT flowchart for selecting and monitoring group participants. Municipality of northeastern Brazil, 2019.

The groups were formed from Basic Health Units (BHUs), where family health teams with a dentist work. These are health services that offer primary healthcare. Random sampling was performed without replacement from a list of 20 BHUs selected for the study using BioEstat version 5.3 (Instituto Mamirauá, Tefé, Brazil) for composition of the two groups; ten BHUs in the intervention group (IG) and ten BHUs in the control group (CG) would follow routine prenatal care (Figure 1). This selected study design avoids the risk of bias from the study experiment, respecting Consort’s recommendation [17].

The participation of pregnant women was conditioned to registration in the public health information system (e-sus) and prenatal follow-up in the selected BHU. The selection criteria were as follows: willingness to participate in the intervention meetings; to be in the first trimester of pregnancy or, at most, in the first prenatal consultation of the second trimester in the recruitment period, which took place from April to June 2018. Exclusion criteria were related to health, including psychiatric problems, HIV, autoimmune diseases and illicit drug use, and to participation, including a lack of two consecutive or three alternate collective activities. Abortion, change of the drawn area or maternal death were characterized as losses.

For the sample calculation, the following parameters were used: 95% confidence interval, frequency of oral diseases in pregnant women of 21% [18], difference of 40% in the frequency of these diseases between the groups after the intervention and a sampling power of 80%. The sample size of pregnant women eligible to participate in the study was 198, divided into two groups. However, the final analysis was performed with 146 pregnant women, with 58 belonging to the intervention group (IG), considering the protocol’s inclusion criteria, and 88 pregnant women for the control group (CG); the losses and reasons are exposed in the flowchart (Figure 1).

Data gathering was performed from April 2018 to January 2019 with the participation of research assistants trained by four nutrition students, four nursing students and two dentists (*n* = 10) to level and apply the pilot test of instruments and collection. The research assistants attended on the days scheduled for prenatal consultation of the IG and CG at the BHU in order to identify the pregnant women who met the requirements at the first moment and to continue the study.

### 2.2. Intervention Description

The integrated care intervention for pregnant women was designed and validated by the consensus of specialists. It consisted of actions of healthcare, promotion and surveillance within the scope of primary care considering the entire gestational period until the beginning of the puerperium.

Pregnant women classified as having intermediate or high risk in oral health received a home visit from a community health worker (CHW) to reinforce the need to attend the BHU for dental treatment and guidance on oral hygiene care. To increase intervention adherence and follow-up, the research assistants made a telephone call to each patient five days before the collective actions (CAs) meeting to confirm the date and emphasize the importance of maintaining follow-up.

The assistance action consisted of a dental treatment plan registered in the medical records and a “warning” on the pregnant woman’s card of the need for dental care at the BHU.

Concomitantly with dental care, in the IG, the pregnant women participated in biweekly meetings for health promotion actions (Cas) using the conversation wheel technique. This technique, which provides a space for dialogue and reflection of health problems, enables integrated healthcare strategies and contributes to health promotion [19,20].

In the conversation wheels, the pregnant woman’s card, vaccines and routine prenatal examinations were observed and questions were asked about complications in the period.

A program was elaborated with 14 Cas, lasting for an average of 60 min each, divided by trimester.

1st trimester of pregnancy: Five CAs were carried out, addressing the following themes: physiological, emotional changes and nutritional risks in pregnancy; harm of drugs (smoking, alcoholic beverages and other legal and illegal drugs); oral hygiene guidance and practice; healthy eating, obesity prevention, diabetes, hypertension and use of sugars and sweeteners; exchange of experiences between pregnant women.

2nd trimester of pregnancy: Four CAs were carried out, covering the following themes: gingival and periodontal disease during pregnancy; the importance of daily personal and oral hygiene; self-examination of the mouth (prevention of oral cancer and other diseases); oral hygiene guidance and practice; food hygiene and rational use of sugar and sweeteners; social and labor rights for pregnant women.

3rd trimester of pregnancy: Five CAs were carried out, addressing the following themes: labor, family planning, exams and baby vaccines; exclusive breastfeeding and baby’s oral health; prevention of caries, oral hygiene and care for baby’s utensils; responsible parenting and accident prevention; oral hygiene guidance and practice.

Every two months, during the intervention period, the main researcher visited the BHU nurses for complementary information about the pregnant women (IG and CG) regarding complications to define the follow-up in the study.

A questionnaire was applied to all participants, from both groups, in a private room or in the BHU dental office.

The questionnaire was composed of five parts: 1—General Identification, including identification of the BHU, data gathering date, name of the participant, address, telephone contact, gestational period and name of the community health agent; 2—Sociodemographic Factors, including maternal age in years, skin color/ethnicity, marital status and how many people live in the house; 3—Socioeconomic Data, including educational level (categorized as <5 years of study, incomplete primary school (from 1st grade to 8th grade), complete primary school (from 9th grade to 2nd grade of high school), incomplete high school and higher education (3rd year of high school and incomplete higher education) and complete higher education); family income based on the minimum wage in force in 2018 (261.36 USD) [21] (≤1 salary, 1–2.9 salaries, ≥3 salaries); home water supply (indoor household water from the public network, piped water from a well or a spring, water from a neighbor or other); 4—Behavioral Factors, including smoking (yes/no); alcohol (yes/no); daily tooth brushing (yes/no); food and access to dental services; 5—Self-perception and impacts on oral health.

To stratify the oral health risk, a clinical examination was carried out in the dental office (using procedure gloves and wooden spatula) of each basic health unit at two points: after the questionnaire was applied at the beginning of the study and during the puerperal visit by auxiliary BHU dentists and the main researcher. The oral health risk of both groups was stratified, which served as a baseline and was included in the medical record. The risk stratification took into account biological criteria (diabetes, hypertension, pregnant woman, use of alcohol and/or tobacco, adolescent) and socioeconomic and dental information (unemployed or not, toothache in the last six months, number of teeth with caries injury, mouth sore, gingival bleeding, need for specialized treatment); each item was assigned a score, the sum of which was evaluated at the end, being classified as low risk (0–10 points), medium risk (11–30 points) and high risk (over 30 points) [22,23]. All participants were informed of their risk classification; pregnant women who were members of the CG were instructed to seek dental care at the BHU and the IG had already started the dental intervention at the BHU.

Information on the occurrence of complications in childbirth and the babies’ data (gestational age, birth weight and complications) were collected in the BHU and/or in the puerperal consultation with the participants of both groups.

Maternal and child factors included the following: prematurity (term ≥37 weeks; preterm <37 weeks); maternal complications in the puerperal pregnancy period—no, with complication (hypertension, gestational diabetes, pre-eclampsia, postpartum hemorrhage); child weight at birth in grams—normal weight ≥2500, low weight <2500; baby complications—without complications, with complications (large for gestational age (LGA), neonatal death).

### 2.3. Analysis Plan

Descriptive analysis and frequency distribution were used to describe the sociodemographic, behavioral, maternal and child variables and oral health risk. Descriptive analyses were performed using the Jasp 13.1 program (Free Version, University of Amsterdam, Amsterdam, The Netherlands).

A network analysis was used to assess the association between sociodemographic, behavioral, maternal and child oral health factors and group (intervention and control). The betweenness, closeness and strength indicators have been reported; variables with higher betweenness values are more sensitive to changes from interventions and can act as a hub, connecting other pairs of variables in the network. A variable with a high proximity value (closeness) will be quickly affected by changes anywhere in the network and can also affect other parts. The strength indicator is essential for understanding which variables have the most robust connections in the current network pattern [24].

The Fruchterman–Reingold algorithm was applied; therefore, the data were shown in the relative space, in which the variables with stronger permanent statistics together and those with less strongly applied variations repelled each other. To improve network accuracy, we used the pairwise Markov random fields model. The algorithm adds a penalty “L1” (regularized neighborhood regression). The adjustment is estimated by a less complete selection and contraction operator (Lasso) that controls the sparse network [25].

Network analysis uses regularized algorithms of least absolute shrinkage and selection operator (LASSO) to obtain the precision matrix, which, when standardized, represents the associations between the variables present in the network. For a better visualization of the weight matrix, the network is presented in a chart that includes the variables (nodes) and relationships (lines). Blue or green color represents positive associations, and red represents negative associations. The thickness and intensity of the colors represent the magnitude of the associations. The R studio Q GRAPH package was used [24].

The extended Bayesian information criterion (EBIC) was observed to select the lambda of the regularization parameter [26]. EBIC uses a hyper parameter (y) that determines how much the EBIC selects sparse models. The value of y was determined as 0.25 (range from 0 to 0.50), which is a more parsimonious value when there are exploratory networks, as in the present study.

The ethical aspects of the Declaration of Helsinki were respected [27]. This study was approved by the Research Ethics Committee at the Integral Medical Institute Professor Fernando Figueira—IMIP/PE, under no. 1.744.599, and the University of Juazeiro do Norte—Unijuazeiro/CE, under no. 1.802.276.

## 3. Results

### 3.1. Descriptive

Descriptive statistics were used to describe sociodemographic and behavioral variables, oral health risk and maternal and newborn complications.

The descriptive analysis indicated that the control group presented a higher frequency of maternal complications (18.8%), baby complications (11.4%), prematurity (18.2%) and low birth weight (13.6%) (Table 1).

Table 2 shows the values of frequency distribution of the initial and final oral health risk according to groups (IG and CG). The results indicate a decrease in the percentage of pregnant women with high-risk oral health; in the IG, there was a change from 29.3% to 12.1%, and in the CG, this change was from 36.4% to 21.6%.

### 3.2. Analysis of Networks (Intervention/Associative)

**Figure 2:**
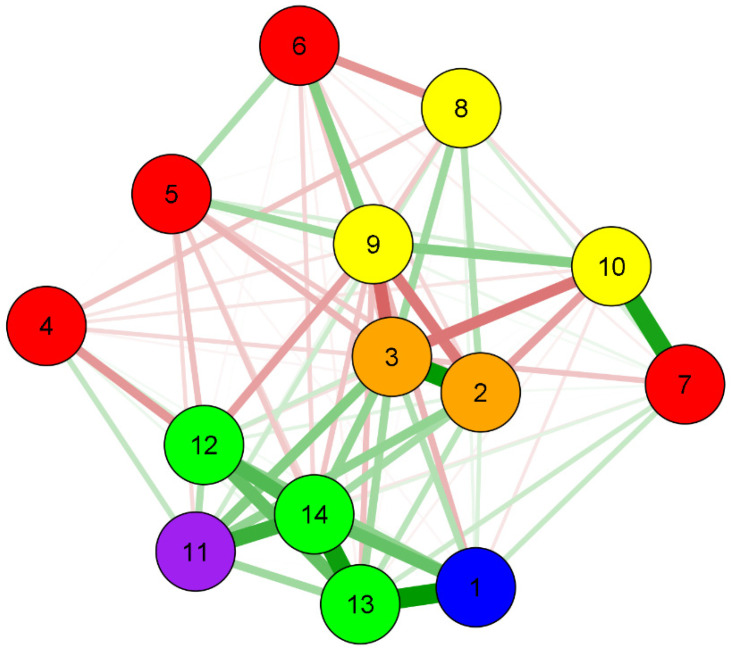
Network perspective of the association between sociodemographic and behavioral factors, group (intervention and control) and mother–child oral health. 1: Group; 2: Initial oral health risk; 3: Final oral health risk; 4: Age; 5: Educational level; 6: Income; 7: Home water; 8: Oral hygiene; 9: Smoking; 10: Alcohol; 11: Maternal complications; 12: Birth weight; 13: Baby’s complications at birth; 14: Prematurity.

The main results of the network analysis indicate that the control group is associated with neonatal complications (0.89) and prematurity (0.54). High-risk initial and final oral health is associated with smoking and alcohol use. From the group analysis, it can be seen that smoking is associated with high-risk initial (−0.47) and final (−0.54) oral health in the control group, and alcohol use is associated with a high risk in final oral health in the control group (Table 3 and Figure 2).

The higher the mother’s age is, the higher the risk of maternal complications (0.21) and prematurity (0.14) is, but the lower the maternal age, the greater the chance is of the newborn having a low birth weight (−0.37). Lower education level of pregnant women is associated with a high risk in initial (−0.19) and final (−0.26) oral health; maternal complications (−0.13); low birth weight (−0.23); complications (−0.14) and prematurity (−0.19). Low family income (≤261.36 USD) is associated with high-risk initial (−0.14) and final (−0.20) oral health; not brushing teeth (−0.37) and prematurity (−0.15). Not having treated and piped water at home is associated with alcohol use (0.81).

### 3.3. Centrality Measures

Table 4 presents the network centrality measures. In the strength indicator, the variables final oral health risk (1.663), baby complications (1.035) and prematurity (1.317) presented the highest values. In the expected influence, the variables of group (0.930), maternal complications (1.084), baby complications (1.534) and prematurity (1.631) presented the highest values, and in the closeness indicator, the variables final oral health risk (1.807) and smoking (1.354) presented the highest values.

## 4. Discussion

This research shows the assessment of the effectiveness of integrated healthcare for pregnant women. The results confirm the hypothesis of the study that pregnant women, fully assisted during prenatal care with dental treatment and attending the collective actions of guidelines, presented better pregnancy outcomes than their counterparts in the control group. In the CG, more cases of prematurity and complications were found in newborns, including neonatal death, as well as high risk in initial and final oral health in pregnant women; this oral condition showed an association with behavioral factors such as smoking and the use of alcohol, suggesting that the intervention was a protective factor.

The positive effect of the intervention highlighted here is similar to that of an Australian study with pregnant women followed up until delivery, which concluded that the pregnant women in the intervention group who received obstetric and dental care and guidance adhered more to the guidelines and oral health treatment than the other groups did that received only guidance on prenatal care and oral health [4]. This adherence of the pregnant women of the IG to the proposed treatment and collective actions was evidenced during the research. They were concerned with booking the date of the next action, and during the meetings, they asked questions about the subject to clarify their doubts, stimulating debate in the group. As a result, although there are few evidence-based oral health promotion interventions for pregnant women, full monitoring with obstetric professionals and dentists for them during prenatal care must be given, ensuring a network of dental services including primary care [4].

This protection can also be attributed to frequent collective actions on healthy habits in the intervention group, where pregnant women were able to expose their habits during the conversation wheels and during the exchange of knowledge, allowing personal reflections, even with motivation to incorporate healthy eating not only for themselves but for everyone in the household, often worrying about the existing younger child [19,20]. Primary care involving oral and systemic healthcare for mothers and their newborns should be guided in interprofessional work with collaboration between educators and health professionals in order to evidence and incorporate oral health needs as the gold standard for educational programs and clinical practice [28]. This result corroborates the findings of another study, explaining that the perinatal period is important to perform interventions because some determinants of general and oral health are established at this stage. Moreover, women are more motivated to adopt healthy behaviors for the fetus [29].

The collective actions developed in this research are some of the themes most often found in a systematic review for health education of pregnant women, highlighting the following: breastfeeding, nutrition, delivery, childcare, family planning, physical activity, maternal health, anxiety, social support, drug abuse, oral health and baby development. In addition to this finding, health education strategies conducted by group methodologies with a qualitative approach and guided by professionals contribute to reducing prematurity, low birth weight and increasing prevalence of exclusive breastfeeding in addition to increasing feelings of self-confidence, safety and calmness among pregnant women [30].

Another point to be highlighted is the high risk in the initial and final oral health of pregnant women with smoking and alcohol use, confirming the results found in alcohol-dependent individuals with a higher prevalence of dental caries, periodontitis and oral mucosa lesions compared to non-alcoholic individuals [31], as well as greater periodontal involvement in smokers [32].

A systematic review with meta-analysis showed a positive association between maternal periodontitis and premature birth, with an OR (Odds Ratio) value of 2.01 (95% CI: 1.71, 2.36). The authors recommended that health and educational services prioritize this risk factor to reduce the incidence of prematurity, implementing actions that favor prevention in all women of childbearing age [33]. Thus, it is important to monitor pregnant women of the intervention group and to provide educational orientations carried out through conversation wheels on these topics, which did not occur in the control group and may have influenced the permanence of these pernicious habits.

When analyzing the variable maternal age, the present study identified that mothers of older ages have a higher risk of maternal complications and prematurity, but the lower the maternal age is, the higher the chances are of having a low birth weight (<2500 g). The results are consistent with a systematic review where it was observed that maternal ages below 20 years and older than 35 years significantly influence low birth weight [34]. This reality reinforces the need to intensify family planning actions early considering the age group of women of childbearing age.

Upon analyzing the social determinants in this study, low family income (less than USD 261.36) was found to be associated with high oral health risk and the chance of not brushing the teeth daily. Likewise, in a study carried out in Brazil with adults, it was found that income is fundamental in the promotion of oral health, where low income is a limiting factor for access to hygiene resources and essential for maintaining a good oral condition. It also showed that an increase in the frequency of daily brushing, use of a brush, toothpaste and dental floss together and replacement of the old brush with a new one (once every three months) increases with income [35]. Another study of cohort trends in Brazil demonstrated a trend of premature births in babies born from mothers with a low income [36]. 

Among the social factors, our findings showed an association between lack of treated and piped water at home and alcohol use, lower educational level and high risk in oral health, maternal and baby complications and low birth weight and prematurity, agreeing with another study whose results indicated that the mother’s lower educational level is, the lower the income is, the poorer the housing situation is and the higher the risk of having general and oral health problems is [37].

In the network analysis, the variables identified as having a greater sensitivity to interventions were maternal and neonatal complications, prematurity and the group variable. These can act as a hub by connecting other pairs of variables in the network. The risk of final oral health and smoking were the variables that presented high proximity to the influenceable variables. This feature causes them to be more rapidly affected by changes in any part of the network, which, in turn, can affect others. The variables that were the strongest in the network were final oral health risk, prematurity and complications of the baby. This strength indicator is essential to understand which variables have the most robust connections in the current network pattern.

Previous studies have indicated that mothers with periodontal disease at the time of delivery had a 3.4 times greater chance of having pre-eclampsia, and their children were 2.6 times more likely to present low birth weight [38]. A review study published in 2015 had already verified that maternal periodontal disease, associated with low socioeconomic conditions, is a potential indicator of independent risk for these adverse events, suggesting that oral care should be part of the prenatal preventive care provided, especially in developing countries [39].

Belonging to the intervention group, educational collective actions on general healthcare and dental treatment and follow-up proved to be a protective factor due to the opportunity to allow chances for behavior changes and, consequently, reduction in maternal complications and prematurity. It is worth mentioning that these actions are related to the health area, while low income and low educational level are also risk factors for negative outcomes, but they are beyond the health scope, depending, instead, on public policies that equalize these disparities.

As this is a community trial, some limitations should be listed, such as external factors that cannot be controlled by researchers—for example, a strike by some health professionals in the municipality, which caused loss of follow-up with pregnant women who went to other health services or cities in order to continue prenatal care, and the profile of some professionals working in primary care who do not establish a professional–patient bond, thus compromising the process of adhering to self-care and assistance actions [40].

## 5. Conclusions

The intervention based on integrated oral health care for pregnant women was effective in relation to positive pregnancy outcomes and oral health. The socioeconomic and behavioral aspects analyzed are emphasized as influencing variables that can be priorities for the health of women and children and that, in linear analyses, do not demonstrate robustness of their strength. The network perspective allows for verifying the complex connection between different variables and the possible influences that they have that modify reality. It is also noteworthy that the involvement of several professionals in prenatal care contributes positively to self-care in health by pregnant women, which is transformed into benefits for future generations and for women’s quality of life.

## Figures and Tables

**Table 1:** Sociodemographic, behavioral, oral and mother–child health characteristics of the sample.

Variables	General (*n* = 146)	Intervention (*n* = 58)	Control (*n* = 88)
	*n* (%)	*n* (%)	*n* (%)
Age (mean and standard deviation)	25.4 (6.7)	25.1 (6.8)	25.6 (6.6)
Initial oral health risk			
Low risk	59 (40.4)	26 (44.8)	33 (34.5)
Intermediate risk	38 (26)	15 (25.8)	23 (26.1)
High risk	49 (33.6)	17 (29.3)	32 (36.3)
Final oral health risk			
Low risk	83 (56.8)	40 (68.9)	43 (48.8)
Intermediate risk	37 (25.3)	11 (18.9)	26 (29.5)
High risk	26 (17.8)	7 (12)	19 (21.5)
Educational level			
<5 years of study	1 (0.68)	0 (0)	1 (1.1)
Incomplete primary education	51 (34.93)	21 (36.2)	30 (34.9)
Complete primary education	27 (18.49)	8 (13.7)	19 (21.5)
Complete high school	58 (39.72)	26 (44.8)	32 (36.3)
Higher education	9 (6.16)	3 (5.3)	6 (6.0)
Family income			
≤1 wage	87 (60.3)	32 (55.2)	55 (62.5)
1–2.9 wages	49 (36.3)	20 (34.0)	29 (33.0)
≥3 wages	10 (3.4)	6 (11.2)	4 (4.5)
Home water supply			
Piped water (public service)	134 (91.8)	55 (94.8)	79 (89.7)
Piped water, well or spring	9 (6.2)	2 (3.4)	7 (7.9)
Water from neighbor	2 (1.4)	1 (1.7)	1 (1.1)
Other	1 (0.7)	0	1 (1.1)
Do you brush your teeth?			
Yes, I brush every day	140 (95.9)	56 (96.5)	84 (95.4)
Yes, but not every day	6 (4.1)	2 (3.4)	4 (4.5)
Smoking			
Yes	13 (8.9)	3 (5.1)	10 (11.3)
No	133 (91.1)	55 (94.8)	78 (88.6)
Alcohol			
Yes	15 (10.3)	5 (93.1)	10 (11.3)
No	131 (89.7)	4 (6.8)	78 (88.6)
Maternal complications (in the postpartum pregnancy period)			
No complications	120 (82.1)	48 (82.8)	72 (81.2)
With complications	26 (17.9)	10 (17.2)	16 (18.8)
Complications of NB * at birth			
No complications	135 (85.5)	57 (98.2)	78 (88.6)
With complications	11 (14.5)	1 (1.8)	10 (11.4)
Prematurity			
<37 preterm	20 (13.7)	4 (6.9)	16(18.2)
>37 term (normal time)	126 (86.3)	54 (93.1)	72(81.8)
Birth weight			
Low weight	14 (9.6)	2 (3.4)	12 (13.6)
Normal	132 (90.4)	56 (96.6)	76 (86.4)

* NB—newborn.

**Table 2:** Frequency distribution of the initial and final oral health risk according to groups (intervention group (IG) and control group (CG)). County in northeast Brazil, 2019.

Oral Health Risk	Low Risk *n* (%)		Intermediate Risk *n* (%)		High Risk *n* (%)	
Groups	Initial	Final	Initial	Final	Initial	Final
Intervention	26 (44.8)	40 (69.0)	15 (25.9)	11 (19.0)	17 (29.3)	7 (12.1)
Control	33 (37.5)	43 (48.9)	23 (26.1)	26 (29.5)	32 (36.4)	19 (21.6)

**Table 3:** Associations between the variables present in the network.

Variables	Group	IOHR *	FOHR *	Age	Educational Level	Income	Home Water	Oral Hygiene	Smoking	Alcohol	Maternal Comp.^#^	Weight	NB Comp.^#^	Prematurity
Group	0.00	0.13	0.27	0.04	−0.03	−0.01	0.20	0.08	−0.26	−0.09	−0.01	0.40	0.89	0.54
IOHR *	0.13	0.00	0.88	−0.04	−0.19	−0.14	0.04	0.27	−0.47	−0.33	0.38	0.10	0.24	0.32
FOHR *	0.27	0.88	0.00	−0.04	−0.26	−0.20	−0.21	0.33	−0.54	−0.48	0.43	0.17	0.33	0.38
Age	0.04	−0.04	−0.04	0.00	0.00	0.01	−0.14	−0.21	−0.10	−0.10	0.21	−0.37	0.07	0.14
Educational level	−0.03	−0.19	−0.26	0.00	0.00	0.29	0.04	0.02	0.33	0.14	−0.13	−0.23	−0.14	−0.19
Income	−0.01	−0.14	−0.20	0.01	0.29	0.00	−0.06	−0.37	0.43	0.02	0.01	−0.03	−0.05	−0.15
Home Water	0.20	0.04	−0.21	−0.14	0.04	−0.06	0.00	0.14	0.07	0.81	−0.08	0.01	0.18	0.10
Oral Hygiene	0.08	0.27	0.33	−0.21	0.02	−0.37	0.14	0.00	−0.19	−0.12	0.17	−0.04	−0.02	−0.01
Smoking	−0.26	−0.47	−0.54	−0.10	0.33	0.43	0.07	−0.19	0.00	0.42	0.07	−0.33	−0.22	−0.21
Alcohol	−0.09	−0.33	−0.48	−0.10	0.14	0.02	0.81	−0.12	0.42	0.00	−0.01	−0.11	−0.05	0.02
Maternal Comp.	−0.01	0.38	0.43	0.21	−0.13	0.01	−0.08	0.17	0.07	−0.01	0.00	0.28	0.33	0.69
Weight	0.40	0.10	0.17	−0.37	−0.23	−0.03	0.01	−0.04	−0.33	−0.11	0.28	0.00	0.55	0.61
NB Comp.	0.89	0.24	0.33	0.07	−0.14	−0.05	0.18	−0.02	−0.22	−0.05	0.33	0.55	0.00	0.85
Prematurity	0.54	0.32	0.38	0.14	−0.19	−0.15	0.10	−0.01	−0.21	0.02	0.69	0.61	0.85	0.00

Notes: Blue represents positive associations and red represents negative associations. The intensity of the colors represents the magnitude of the associations. * IOHR and FOHR—initial and final oral health risk, respectively; ^#^ Maternal Comp.—maternal complications; NB Comp.—neonatal complications (NB—newborn).

**Table 4:** Network centrality measures.

Variables	Closeness	Strength	Expected Influence
Group	−0.107	0.039	0.930
Initial oral health risk	0.721	0.660	0.202
Final oral health risk	1.807	1.663	0.101
Age	−1.476	−1.494	−1.094
Educational level	−1.109	−0.959	−0.976
Income	−0.956	−1.183	−0.895
Home water	−0.939	−0.854	0.140
Oral hygiene	−1.052	−0.984	−0.658
Smoking	1.354	0.761	−1.454
Alcohol	−0.113	−0.210	−0.613
Maternal complications	0.330	−0.118	1.084
Birth weight	0.335	0.326	0.068
Baby complications	0.376	1.035	1.534
Prematurity	0.829	1.317	1.631

## Data Availability

Not applicable.
